# Anatomy-guided context-aware deep learning for lumbar degenerative disease grading and burden-aware risk assessment on MRI

**DOI:** 10.3389/fmed.2026.1848548

**Published:** 2026-06-26

**Authors:** Zhijin Chai, Chen Liu, Rujie Qin, Dexuan Zhao, Ankang Shi

**Affiliations:** 1 Lianyungang Clinical College of Nanjing Medical University, Lianyungang, Jiangsu, China; 2 The Affiliated Lianyungang Hospital of Xuzhou Medical University, Lianyungang, Jiangsu, China

**Keywords:** anatomy-guided deep learning, degenerative spine disease, interpretability, lumbar spine MRI, multi-level context modeling, quantitative biomarkers, risk assessment, spinal stenosis grading

## Abstract

Automated lumbar spine MRI analysis has made notable progress in recent years, yet existing methods still suffer from anatomical ambiguity, insufficient use of explicit structural priors, and limited modeling of inter-level degenerative dependency, which restrict their reliability for clinically meaningful burden assessment. To address these limitations, we propose an anatomy-guided, multi-sequence, multi-level deep learning framework for lumbar degenerative disease grading and patient-level risk assessment. First, an anatomical parsing module is pre-trained to segment vertebral bodies, intervertebral discs, and the spinal canal, providing stable level localization and structural priors for downstream analysis. Then, the localized multi-sequence MRI patches and quantitative anatomical biomarkers are jointly encoded and further modeled by a lightweight Transformer to capture contextual dependency across lumbar levels, enabling both segment-level ordinal grading and Clinically Significant Degeneration Score (CSDS)-based patient-level burden assessment. Extensive experiments on public datasets demonstrate that the proposed framework achieves superior performance over representative baselines, reaching a Macro F1-score of 0.783 ± 0.010, a Cohen's Kappa of 0.765 ± 0.012, a weighted log loss of 0.463 ± 0.018, and a patient-level AUC of 0.891 ± 0.009, while supplementary evaluations further verify the robustness of the anatomical parsing stage. The ablation results show that anatomy-guided cropping, quantitative biomarker fusion, spine-context modeling, and consistency regularization each contribute complementary gains, confirming that the effectiveness of the framework arises from their coordinated interaction rather than from any single component alone. Overall, these findings indicate that explicitly integrating anatomical priors, structured biomarkers, and multilevel context can substantially improve both diagnostic accuracy and interpretability, while also supporting more standardized, transparent, and human-centered lumbar MRI assessment in radiological practice.

## 1 Introduction

Degenerative disorders of the lumbar spine are among the most common imaging-visible causes of low back pain, radicular symptoms, and functional limitation in adults. Lumbar magnetic resonance imaging (MRI) is the primary modality for assessing spinal canal stenosis, neural foraminal narrowing, sub-articular stenosis, and related multilevel degenerative changes. Recent systematic evidence indicates that artificial intelligence and deep learning have shown increasing promise for improving the diagnosis of lumbar spinal stenosis and related disorders, but their clinical translation still depends on robustness, interpretability, and workflow compatibility rather than accuracy alone (1–4).

Over the past several years, substantial progress has been made in automated lumbar MRI analysis. Earlier studies such as SpineNet demonstrated that deep neural networks could learn radiological grading patterns directly from lumbar MRI and provide lesion evidence visualization (5). Subsequent external validation studies further supported the feasibility of automated degenerative grading across clinical cohorts (6, 7). More recently, task-specific studies have reported encouraging performance for lumbar stenosis and related degenerative findings (2, 3, 8). In parallel, segmentation-oriented studies have improved the anatomical delineation of lumbar structures and strengthened the feasibility of anatomy-guided preprocessing for downstream grading tasks (9, 10). At the same time, the release of public resources such as SPIDER, LumbarDISC, and open-access lumbosacral MRI datasets has substantially improved the reproducibility and comparability of lumbar spine imaging research (9, 11–14).

Despite these advances, three key limitations remain insufficiently resolved. First, many existing methods still rely on whole-image classification or loosely aligned local patches, making them vulnerable to anatomical ambiguity and inter-subject structural variation (2, 3, 8). Second, most prior studies focus on isolated segment-level assessment while underutilizing explicit anatomical priors and quantitative geometric biomarkers that are clinically meaningful for radiological interpretation (2, 3, 8). Third, adjacent lumbar levels are biomechanically and clinically interdependent, yet cross-level contextual modeling is still underexplored in most lumbar MRI grading pipelines (5, 6, 8). As a result, existing systems often perform well at local classification but remain weaker at transparent and clinically coherent burden estimation.

To address these issues, we propose an anatomy-guided, multi-sequence, multi-level deep learning framework for lumbar degenerative disease grading and burden-aware patient-level risk assessment. The framework integrates three tightly coupled stages: anatomy-guided structural parsing for level localization, multi-branch segment-level representation learning from both MRI appearance and quantitative biomarkers, and spine-context encoding for modeling inter-level dependency. Specifically, a Stage I anatomical parser is initialized using VerSe and refined on SPIDER to generate vertebral, disc, and canal masks for stable lumbar level indexing (9, 15). The refined parser is then transferred to LumbarDISC to support level-specific ROI extraction and biomarker computation, after which local image features, biomarker embeddings, and ordered lumbar context are jointly modeled for segment-level ordinal grading and patient-level CSDS-based assessment (11–13).

The proposed design is motivated by both radiological reasoning and human-centered AI principles. In routine practice, radiologists first identify the correct lumbar level, then assess local structural abnormalities, and finally integrate multilevel findings into an overall estimate of disease burden. Our framework mirrors this process through explicit anatomical localization, measurable structural biomarkers, and context-aware multilevel aggregation. This design improves not only diagnostic performance, but also interpretability through mask visualization, biomarker transparency, Grad-CAM explanation, and level-wise attention. Such properties are especially important for clinically transparent decision support, structured reporting, and more standardized radiological assessment, which aligns with broader recommendations for trustworthy radiology AI and clinically deployable medical machine learning (16–19).

Extensive experiments on public datasets demonstrate that the proposed framework consistently outperforms representative re-implemented baselines on LumbarDISC, achieving superior segment-level grading and patient-level discrimination while maintaining clear interpretability. These findings suggest that anatomy-aware and burden-aware deep learning offers a promising direction for lumbar MRI analysis, especially for scenarios in which transparent assistance, standardized grading, and clinically meaningful multilevel reasoning are required.

In summary, the main contributions of this work are threefold. **First**, we propose an anatomy-guided multi-stage framework that explicitly connects structural parsing, level-aligned ROI extraction, and degenerative grading within a unified pipeline. **Second**, we introduce a burden-aware modeling strategy that combines local MRI appearance, structured anatomical biomarkers, and inter-level sequence context, enabling both segment-level ordinal grading and patient-level CSDS-based burden assessment. **Third**, extensive experiments on public datasets demonstrate that the proposed framework achieves superior performance over representative baselines while preserving clinically meaningful interpretability, highlighting its potential value for transparent and scalable lumbar spine MRI analysis.

## 2 Method

### 2.1 Network architecture

#### 2.1.1 Stage I: anatomy-guided structural parsing and level localization

**Figure 1:**
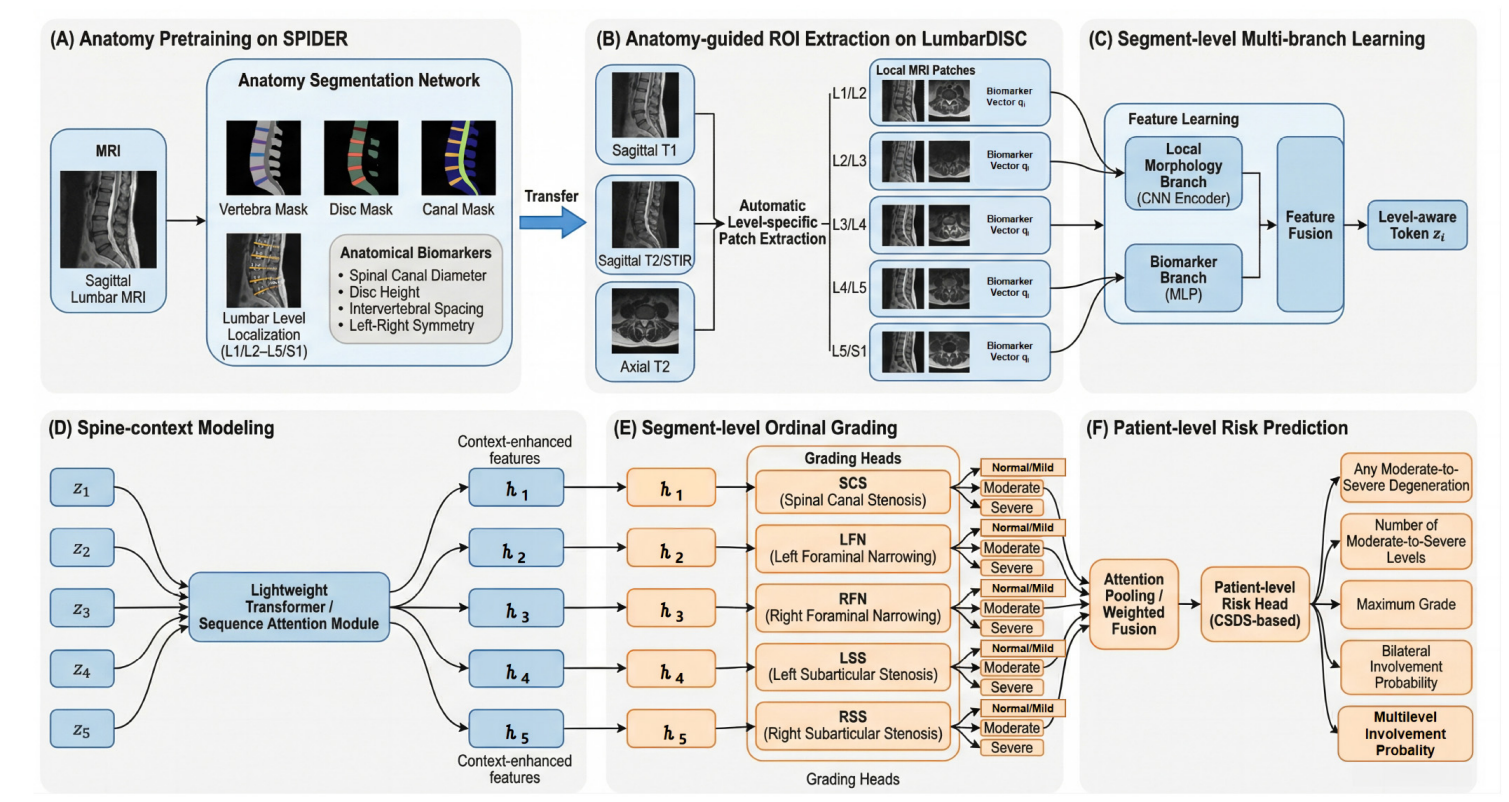
Overview of the proposed anatomy-guided and context-aware framework for lumbar degenerative disease grading and patient-level risk assessment on MRI. **(A)** On the SPIDER dataset, an anatomy segmentation network is first trained to segment vertebral bodies, intervertebral discs, and the spinal canal, from which lumbar level localization and quantitative anatomical biomarkers are derived. **(B)** The pretrained anatomical parser is transferred to LumbarDISC to guide automatic extraction of level-specific multi-sequence MRI patches and biomarker vectors for each lumbar motion segment from L1/L2 to L5/S1. **(C)** For each lumbar level, a segment-level multi-branch learning module combines a local morphology branch for MRI appearance encoding and a biomarker branch for structured anatomical measurements, followed by feature fusion to generate a level-aware token. **(D)** The ordered level-aware tokens are then fed into a lightweight Transformer or sequence attention module to model inter-level contextual dependency and produce context-enhanced representations. **(E)** Based on these context-enhanced features, segment-level ordinal grading is performed for spinal canal stenosis, left/right neural foraminal narrowing, and left/right subarticular stenosis. **(F)** Finally, the segment-level outputs are aggregated through attention pooling or weighted fusion to produce a patient-level CSDS assessment head, which estimates the Clinically Significant Degeneration Score (CSDS)-based burden profile, including any moderate-to-severe degeneration, number of moderate-to-severe levels, maximum grade, bilateral involvement probability, and multilevel involvement probability.

As shown in Figure 1, the proposed framework takes lumbar MRI as input and first performs anatomy-guided structural parsing to establish reliable level-wise localization. Let the sagittal MRI volume be denoted as

$${\text{X}}^{\text{sag}}\in {\mathbb{R}}^{H_{s}\times W_{s}\times D_{s}},$$

and the axial MRI volume be denoted as

$${\text{X}}^{\text{ax}}\in {\mathbb{R}}^{H_{a}\times W_{a}\times D_{a}},$$

where *H*, *W*, and *D* denote the image height, width, and slice number, respectively. A segmentation backbone is first applied to **X**^sag^ to obtain the masks of vertebral bodies, intervertebral discs, and the spinal canal:

$$\text{M}=\left\{{\text{M}}^{v},{\text{M}}^{d},{\text{M}}^{c}\right\}.$$

Here, **M**^*v*^, **M**^*d*^, and **M**^*c*^ represent the vertebral, disc, and canal masks, respectively. Based on these structural masks, the centroids of vertebrae and discs are extracted to automatically index the lumbar motion segments from L1/L2 to L5/S1.

For each lumbar level *i*, a level-specific region of interest (ROI) is cropped from the axial MRI:

$${\text{R}}_{i}=\text{Crop}\left({\text{X}}^{\text{ax}},{\text{c}}_{i},h,w,d\right),\;i=1,\ldots ,N,$$

where **c**_*i*_ denotes the level center derived from the disc centroid and canal centerline, (*h, w, d*) denotes the fixed ROI size, and *N* = 5 is the number of lumbar motion segments. Thus,

$${\text{R}}_{i}\in {\mathbb{R}}^{h\times w\times d}.$$

Meanwhile, a set of quantitative anatomical descriptors is extracted from the segmentation masks, including spinal canal diameter, disc height, relative intervertebral spacing, and symmetry-aware geometric measurements. These descriptors are organized as a structured biomarker vector:

$${\text{q}}_{i}={\left[a_{i}^{\left(1\right)},a_{i}^{\left(2\right)},\ldots ,a_{i}^{\left(d_{q}\right)}\right]}^{⊤}\in {\mathbb{R}}^{d_{q}},$$

where *d*_*q*_ is the dimensionality of the anatomical biomarker space.

#### 2.1.2 Quantitative anatomical biomarker extraction from segmentation masks

To make the biomarker branch reproducible and clinically interpretable, the structural descriptors in **q**_*i*_ were derived using deterministic geometric measurements from the predicted vertebral, disc, and canal masks. Let (*s*_*x*_, *s*_*y*_, *s*_*z*_) denote the voxel spacing after resampling, let *z*_*i*_ be the axial slice closest to the disc centroid of level *i*, and let Z_*i*_ denote the small axial slice set covered by the level-specific ROI. For a binary mask **M**, S(**M**, *z*) = {(*u, v*):**M**(*u, v, z*) = 1} denotes the foreground pixels on axial slice *z*.

The spinal canal cross-sectional area was computed as

$$A_{i}^{\text{canal}}=s_{x}s_{y}|S\left({\text{M}}^{c},z_{i}\right)|.$$

The axial anterior-posterior canal diameter was defined as the minimum physical anterior-posterior extent of the canal mask within the level ROI:

$$d_{i}^{\text{AP}}=\underset{z\in Z_{i}}{\min}s_{y}\left(\underset{\left(u,v\right)\in S\left({\text{M}}^{c},z\right)}{\max}v-\underset{\left(u,v\right)\in S\left({\text{M}}^{c},z\right)}{\min}v+1\right),$$

and the transverse canal diameter was computed analogously as

$$d_{i}^{\text{LR}}=\underset{z\in Z_{i}}{\min}s_{x}\left(\underset{\left(u,v\right)\in S\left({\text{M}}^{c},z\right)}{\max}u-\underset{\left(u,v\right)\in S\left({\text{M}}^{c},z\right)}{\min}u+1\right).$$

Thus, canal narrowing is represented by both an area descriptor and a conservative minimum-diameter descriptor rather than by an unspecified single scalar.

For the intervertebral disc mask ${\text{M}}_{i}^{d}$, disc height was measured on the mid-sagittal plane by averaging anterior, central, and posterior distances between the superior and inferior disc boundaries:

$$h_{i}^{\text{disc}}=\frac{1}{3}\underset{r\in \left\{0.25,0.50,0.75\right\}}{\sum }s_{y}\left(y_{i,r}^{\text{sup}}-y_{i,r}^{\text{inf}}\right),$$

where *r* denotes the normalized anterior-posterior sampling position along the disc and $y_{i,r}^{\text{sup}}$ and $y_{i,r}^{\text{inf}}$ are boundary coordinates estimated from the disc mask. To reduce inter-subject size dependence, relative intervertebral spacing was normalized by the mean height of the two adjacent vertebral bodies:

$$\rho_{i}^{\text{space}}=\frac{h_{i}^{\text{disc}}}{0.5\left(h_{i}^{\text{supvert}}+h_{i}^{\text{infvert}}\right)+\epsilon },$$

where ϵ is a small constant for numerical stability.

Left-right symmetry descriptors were obtained by splitting the canal and disc masks at the sagittal midline passing through the canal centroid. For example, canal asymmetry was defined as

$$\Delta_{i}^{\text{canal}}=\frac{\left|A_{i,L}^{\text{canal}}-A_{i,R}^{\text{canal}}\right|}{A_{i,L}^{\text{canal}}+A_{i,R}^{\text{canal}}+\epsilon },$$

and disc-height asymmetry was defined as

$$\Delta_{i}^{\text{disc}}=\frac{\left|h_{i,L}^{\text{disc}}-h_{i,R}^{\text{disc}}\right|}{h_{i,L}^{\text{disc}}+h_{i,R}^{\text{disc}}+\epsilon }.$$

The final biomarker vector was therefore

$${\text{q}}_{i}={\left[A_{i}^{\text{canal}},d_{i}^{\text{AP}},d_{i}^{\text{LR}},h_{i}^{\text{disc}},\rho_{i}^{\text{space}},\Delta_{i}^{\text{canal}},\Delta_{i}^{\text{disc}}\right]}^{⊤},$$

followed by *z*-score normalization using the mean and standard deviation of the training fold only.

#### 2.1.3 Stage II: multi-branch segment-level representation learning

Each level-specific ROI **R**_*i*_ is fed into a local morphology encoder to extract lesion-sensitive image features:

$${\text{f}}_{i}^{\text{loc}}=\Phi_{\text{loc}}\left({\text{R}}_{i}\right),\;{\text{f}}_{i}^{\text{loc}}\in {\mathbb{R}}^{C_{l}},$$

where Φ_loc_(·) denotes the local CNN-based encoder and *C*_*l*_ is the local feature dimension. In parallel, the quantitative biomarker vector **q**_*i*_ is mapped to a latent embedding by a lightweight multilayer perceptron:

$${\text{f}}_{i}^{\text{bio}}=\Phi_{\text{bio}}\left({\text{q}}_{i}\right),\;{\text{f}}_{i}^{\text{bio}}\in {\mathbb{R}}^{C_{b}},$$

where Φ_bio_(·) denotes the biomarker encoder and *C*_*b*_ is the biomarker feature dimension.

The two feature streams are concatenated and projected to obtain a unified level-aware token:

$${\text{z}}_{i}=\text{Proj}\left(\left[{\text{f}}_{i}^{\text{loc}}||{\text{f}}_{i}^{\text{bio}}\right]\right),\;{\text{z}}_{i}\in {\mathbb{R}}^{C_{z}},$$

where || denotes concatenation and *C*_*z*_ is the token dimension. Stacking all lumbar level tokens yields

$$\text{Z}=\left[{\text{z}}_{1},{\text{z}}_{2},\ldots ,{\text{z}}_{N}\right]\in {\mathbb{R}}^{N\times C_{z}}.$$

#### 2.1.4 Stage III: spine-context encoding and dual-level assessment

To capture anatomical continuity and cross-level degenerative dependency, the token sequence **Z** is fed into a context encoder with positional embeddings:

$$\text{H}=\Phi_{\text{ctx}}\left(\text{Z}+\text{P}\right),$$

where $\text{P}\in {\mathbb{R}}^{N\times C_{z}}$ denotes the positional embedding matrix and

$$\text{H}=\left[{\text{h}}_{1},{\text{h}}_{2},\ldots ,{\text{h}}_{N}\right]\in {\mathbb{R}}^{N\times C_{z}}$$

is the context-enhanced level representation.

Based on **H**, a multi-task ordinal grading head estimates the severity of three degenerative findings at each lumbar level:

$${\hat{\text{y}}}_{i}^{\left(t\right)}={\text{Ψ}}_{t}\left({\text{h}}_{i}\right),\;t\in \left\{1,2,3\right\},$$

where *t* = 1, 2, and 3 correspond to spinal canal stenosis, sub-articular recess stenosis, and neural foraminal stenosis, respectively.

To further support clinically meaningful patient-level assessment, the level-wise contextual features are aggregated by attention pooling:

$$\text{g}=\overset{N}{\underset{i=1}{\sum }}\alpha_{i}{\text{h}}_{i},\;\overset{N}{\underset{i=1}{\sum }}\alpha_{i}=1,$$

where α_*i*_ denotes the learned contribution weight of lumbar level *i*. The pooled feature **g** is then passed through a patient-level assessment head to estimate the clinically significant degeneration score:

$$ĉ={\text{Ψ}}_{\text{CSDS}}\left(\text{g}\right),\;ĉ\in \left[0,1\right].$$

In this way, the proposed framework jointly outputs segment-level degenerative grading and patient-level clinically significant degeneration burden.

### 2.2 Innovation method I: anatomy-guided level-aware representation learning

A major limitation of many existing lumbar MRI methods is that they often rely on direct whole-image classification or loosely defined local patches. Such designs may suffer from three issues. First, the network may attend to irrelevant background structures rather than truly disease-related regions. Second, the discriminative boundary between adjacent lumbar levels may become ambiguous when multilevel degeneration is present. Third, purely image-driven classification lacks explicit anatomical constraints, which weakens interpretability and clinical trustworthiness.

To address these limitations, we propose an anatomy-guided level-aware representation learning strategy. Instead of directly classifying the entire image, the proposed framework first decomposes the lumbar MRI into anatomically aligned motion segments. The segmentation module provides vertebral, disc, and canal masks, which are further used to determine the level centers **c**_*i*_ and construct anatomically standardized ROIs **R**_*i*_. Thus, for each subject, the network receives anatomically corresponding inputs for L1/L2 to L5/S1, substantially reducing inter-subject structural variation.

In addition, the extracted biomarker vector **q**_*i*_ provides explicit quantitative structural evidence complementary to the latent image representation. Specifically, the local morphology encoder focuses on implicit texture and shape cues, whereas the biomarker branch preserves measurable anatomical information. Their fusion forms the level-aware token

$${\text{z}}_{i}=\text{Proj}\left(\left[{\text{f}}_{i}^{\text{loc}}||{\text{f}}_{i}^{\text{bio}}\right]\right),$$

which serves as the basic disease-aware representation for lumbar level *i*.

A possible concern is that segmentation errors may propagate to the downstream classifier. To alleviate this issue, the anatomical branch is used as a soft structural prior rather than a hard decision gate. Even when segmentation is imperfect, the local encoder still directly analyzes the raw ROI appearance, and the final assessment is made based on the fused representation. Therefore, the proposed design maintains robustness while improving anatomical consistency and interpretability.

### 2.3 Innovation method II: spine-context-aware multi-task burden assessment

Another critical challenge in lumbar degenerative disease analysis is that disease severity is not determined by a single isolated segment alone. In clinical practice, adjacent levels often exhibit correlated degeneration patterns, and the overall patient burden depends not only on the most severe lesion but also on multilevel involvement. If each level is classified independently, the model may ignore anatomical continuity and underestimate clinically meaningful disease burden.

To address this issue, we propose a spine-context-aware multi-task burden assessment strategy. After obtaining level-wise tokens **z**_*i*_, the model arranges them in lumbar anatomical order to form

$$\text{Z}\in {\mathbb{R}}^{N\times C_{z}},$$

which is further refined by the context encoder:

$$\text{H}=\Phi_{\text{ctx}}\left(\text{Z}+\text{P}\right).$$

The resulting token **h**_*i*_ encodes both local disease evidence and cross-level contextual information, making it more suitable for segment-level grading under multilevel degenerative conditions.

#### 2.3.1 Explicit definition of the clinically significant degeneration score

Because the public datasets used in this study provide cross-sectional level-wise grading labels rather than standardized longitudinal follow-up endpoints, the patient-level assessment target is defined as a *severity-aware clinically significant degeneration score* rather than unsupported future progression probability.

For subject *n*, let the ground-truth grade of task *t* at lumbar level *i* be

$$y_{n,i}^{\left(t\right)}\in \left\{0,1,\ldots ,K_{t}-1\right\},$$

where *K*_*t*_ is the number of ordinal categories for task *t*. We first define the normalized severity at each level as

$$s_{n,i}^{\left(t\right)}=\frac{y_{n,i}^{\left(t\right)}}{K_{t}-1},\;s_{n,i}^{\left(t\right)}\in \left[0,1\right].$$

To emphasize that different degenerative findings and lumbar levels may have different clinical importance, we introduce task weights ω_*t*_>0 and level weights η_*i*_>0. The weighted severity burden of subject *n* is then defined as

$$B_{n}^{\text{sev}}=\frac{1}{\Omega }\overset{N}{\underset{i=1}{\sum }}\overset{T}{\underset{t=1}{\sum }}\omega_{t}\eta_{i}s_{n,i}^{\left(t\right)},\;\Omega =\overset{N}{\underset{i=1}{\sum }}\overset{T}{\underset{t=1}{\sum }}\omega_{t}\eta_{i},$$

where *T* = 3 in this study.

However, severity burden alone does not fully reflect multilevel disease involvement. Therefore, we additionally define a clinically significant lesion indicator:

$$m_{n,i}^{\left(t\right)}=I\left(y_{n,i}^{\left(t\right)}\geq \delta_{t}\right),$$

where I(·) is the indicator function and δ_*t*_ is the task-specific threshold for clinically significant degeneration, typically corresponding to moderate-or-above disease. Based on this, the multilevel involvement burden is defined as

$$B_{n}^{\text{mul}}=\frac{1}{TN}\overset{N}{\underset{i=1}{\sum }}\overset{T}{\underset{t=1}{\sum }}m_{n,i}^{\left(t\right)}.$$

Finally, the ground-truth Clinically Significant Degeneration Score (CSDS) is defined as

$$c_{n}=\lambda_{s}B_{n}^{\text{sev}}+\lambda_{m}B_{n}^{\text{mul}},\;\lambda_{s}+\lambda_{m}=1,\;c_{n}\in \left[0,1\right],$$

where λ_*s*_ and λ_*m*_ are nonnegative coefficients controlling the relative contribution of severity burden and multilevel burden.

For binary patient-level clinically significant burden assessment, we further define the clinically significant degeneration label as

$$r_{n}=I\left(c_{n}\geq \tau \right),$$

where τ∈(0, 1) is the clinically or validation-set selected threshold. Thus, the patient-level target remains fully grounded in the available segment-level annotations.

#### 2.3.2 Context-aware patient-level CSDS assessment

The context-enhanced lumbar features are aggregated to produce the patient representation

$${\text{g}}_{n}=\overset{N}{\underset{i=1}{\sum }}\alpha_{n,i}{\text{h}}_{n,i},$$

which is then mapped to the estimated CSDS:

$${ĉ}_{n}={\text{Ψ}}_{\text{CSDS}}\left({\text{g}}_{n}\right),\;{ĉ}_{n}\in \left[0,1\right].$$

At inference time, the patient is classified as clinically significant degeneration if

$${\hat{r}}_{n}=I\left({ĉ}_{n}\geq \tau \right).$$

This formulation is explicitly cross-sectional. The model estimates the current CSDS-derived burden category from contemporaneous MRI findings and does not forecast future surgery, future symptom progression, treatment response, or longitudinal disease evolution.

### 2.4 Ordinal grading formulation and objective functions

#### 2.4.1 Ordinal formulation for segment-level grading

Each degenerative task is modeled as an ordinal classification problem. For task *t* with *K*_*t*_ ordered categories, the model predicts *K*_*t*_−1 cumulative logits for each lumbar level:

$$a_{n,i,k}^{\left(t\right)}={\text{w}}_{k}^{\left(t\right)⊤}{\text{h}}_{n,i}+b_{k}^{\left(t\right)},\;k=0,1,\ldots ,K_{t}-2.$$

The corresponding cumulative probability is defined as

$$p_{n,i,k}^{\left(t\right)}=\sigma \left(a_{n,i,k}^{\left(t\right)}\right)=P\left(y_{n,i}^{\left(t\right)}>k|{\text{h}}_{n,i}\right),$$

where σ(·) is the sigmoid function.

For each threshold *k*, the binary ordinal target is

$$b_{n,i,k}^{\left(t\right)}=I\left(y_{n,i}^{\left(t\right)}>k\right).$$

Accordingly, the cumulative ordinal loss for task *t* is defined as

$$\begin{array}{l} L_{\text{ord}}^{\left(t\right)}=-\frac{1}{|B|N\left(K_{t}-1\right)}\underset{n\in B}{\sum }\overset{N}{\underset{i=1}{\sum }}\overset{K_{t}-2}{\underset{k=0}{\sum }}\\ {}[w_{t,k}^{+}b_{n,i,k}^{\left(t\right)}\log p_{n,i,k}^{\left(t\right)}+w_{t,k}^{-}1-b_{n,i,k}^{\left(t\right)}\log1-p_{n,i,k}^{\left(t\right)}], \end{array}$$

where B denotes the mini-batch, and $w_{t,k}^{+}$ and $w_{t,k}^{-}$ are positive and negative class-balancing weights for task *t* and threshold *k*.

The total segment-level grading loss is

$$L_{\text{grade}}=\overset{T}{\underset{t=1}{\sum }}\mu_{t}L_{\text{ord}}^{\left(t\right)},$$

where μ_*t*_ is the task weight.

#### 2.4.2 Class probability reconstruction and expected severity

For CSDS aggregation and consistency modeling, we reconstruct the class probabilities from the cumulative probabilities. For task *t*, the class probabilities are defined as

$$\pi_{n,i,0}^{\left(t\right)}=1-p_{n,i,0}^{\left(t\right)},$$

$$\pi_{n,i,k}^{\left(t\right)}=p_{n,i,k-1}^{\left(t\right)}-p_{n,i,k}^{\left(t\right)},\;k=1,\ldots ,K_{t}-2,$$

$$\pi_{n,i,K_{t}-1}^{\left(t\right)}=p_{n,i,K_{t}-2}^{\left(t\right)}.$$

$$\overset{K_{t}-1}{\underset{k=0}{\sum }}\pi_{n,i,k}^{\left(t\right)}=1.$$

The expected grade and expected normalized severity are defined as

$${ȳ}_{n,i}^{\left(t\right)}=\overset{K_{t}-1}{\underset{k=0}{\sum }}k\pi_{n,i,k}^{\left(t\right)},$$

$${\bar{s}}_{n,i}^{\left(t\right)}=\frac{{ȳ}_{n,i}^{\left(t\right)}}{K_{t}-1}.$$

Similarly, the soft probability of clinically significant degeneration at level *i* for task *t* is

$${\bar{m}}_{n,i}^{\left(t\right)}=\overset{K_{t}-1}{\underset{k=\delta_{t}}{\sum }}\pi_{n,i,k}^{\left(t\right)}.$$

#### 2.4.3 Differentiable CSDS aggregation from segment-level estimates

Using the soft severity and soft clinically significant probabilities, we define the differentiable patient-level burden induced by the segment-level outputs:

$${\tilde{B}}_{n}^{\text{sev}}=\frac{1}{\Omega }\overset{N}{\underset{i=1}{\sum }}\overset{T}{\underset{t=1}{\sum }}\omega_{t}\eta_{i}{\bar{s}}_{n,i}^{\left(t\right)},$$

$${\tilde{B}}_{n}^{\text{mul}}=\frac{1}{TN}\overset{N}{\underset{i=1}{\sum }}\overset{T}{\underset{t=1}{\sum }}{\bar{m}}_{n,i}^{\left(t\right)}.$$

The soft CSDS implied by the segment-level outputs is then

$${\tilde{c}}_{n}=\lambda_{s}{\tilde{B}}_{n}^{\text{sev}}+\lambda_{m}{\tilde{B}}_{n}^{\text{mul}}.$$

#### 2.4.4 Patient-level CSDS assessment loss and consistency loss

The patient-level head directly estimates ĉ_*n*_, which is supervised by both the continuous CSDS target *c*_*n*_ and the binary clinically significant degeneration label *r*_*n*_. The patient-level loss is defined as

$$\begin{array}{l} L_{\text{CSDS}}=\lambda_{\text{reg}}\frac{1}{\left|B\right|}\underset{n\in B}{\sum }{\left({ĉ}_{n}-c_{n}\right)}^{2}+\lambda_{\text{cls}}\\ \frac{1}{\left|B\right|}\underset{n\in B}{\sum }[-r_{n}\log{ĉ}_{n}-\left(1-r_{n}\right)\log\left(1-{ĉ}_{n}\right)], \end{array}$$

where λ_reg_ and λ_cls_ are balancing coefficients.

To ensure that the patient-level assessment remains consistent with the segment-level evidence, we introduce a consistency loss:

$$L_{\text{cons}}=\frac{1}{\left|ℬ\right|}\sum_{n\in ℬ}{\left\|{\hat{c}}_{n}-{\tilde{c}}_{n}\right\|}_{2}^{2}.$$

This term prevents the patient-level assessment from becoming an ungrounded black-box score and explicitly couples the global degeneration burden with the level-wise grading outputs.

#### 2.4.5 Overall training objective

The anatomical segmentation module is supervised by a hybrid Dice and cross-entropy loss:

$$L_{\text{seg}}=\lambda_{d}L_{\text{Dice}}+\lambda_{c}L_{\text{CE}}.$$

The overall training objective of the proposed framework is therefore

$$L=\alpha L_{\text{seg}}+\beta L_{\text{grade}}+\gamma L_{\text{CSDS}}+\delta L_{\text{cons}},$$

where α, β, γ, and δ are nonnegative trade-off coefficients. Through joint optimization, the framework simultaneously learns anatomy-guided localization, segment-level ordinal grading, and patient-level clinically significant degeneration assessment in a unified and logically consistent manner.

For reproducibility, all reported experiments used fixed trade-off coefficients

α=1.0,  β=2.0,  γ=1.0,  δ=0.5.

These values were selected using validation-fold performance rather than test-fold results. Specifically, we performed a small validation-only grid search with α, β, γ∈{0.5, 1.0, 2.0} and δ∈{0, 0.5, 1.0}, prioritizing the primary grading objectives while monitoring CSDS calibration and segmentation stability. The final setting assigned a larger weight to *L*_grade_ because segment-level ordinal grading is the main supervised task, retained unit weights for the anatomical and patient-level terms, and used a moderate consistency weight to regularize patient-level aggregation without overwhelming the directly supervised losses. After selection, the coefficients were kept unchanged across all folds and all reported evaluations.

### 2.5 Illustrative case analysis of the CSDS formulation

To provide an intuitive interpretation of the proposed *Clinically Significant Degeneration Score* (CSDS), we present three representative hypothetical cases under a simplified demonstration setting. For ease of exposition, the patient-level score is illustrated using **three pathology categories**—spinal canal stenosis, subarticular stenosis, and neural foraminal narrowing—with left/right laterality merged. Therefore, the number of pathology tasks is set to *T* = 3, and the number of lumbar motion segments is set to *N* = 5 (from L1/L2 to L5/S1). Each task is formulated as a three-grade ordinal problem, namely *K*_*t*_ = 3, where grade 0 denotes normal/mild, grade 1 denotes moderate, and grade 2 denotes severe degeneration.

For each level *i* and task *t*, the normalized severity is defined as


$$s_{n,i}^{\left(t\right)}=\frac{y_{n,i}^{\left(t\right)}}{2},$$

such that grade 0 corresponds to 0, grade 1 corresponds to 0.5, and grade 2 corresponds to 1.0. A task-specific lesion is considered clinically significant when its grade is moderate or above. Accordingly, the binary indicator is defined as


$$m_{n,i}^{\left(t\right)}=I\left(y_{n,i}^{\left(t\right)}\geq \delta_{t}\right),\;\delta_{t}=1.$$

For this illustrative analysis, all task and segment weights are assumed to be equal, i.e.,


$$\omega_{t}=1,\;\eta_{i}=1,$$

which gives


$$\Omega =\overset{N}{\underset{i=1}{\sum }}\overset{T}{\underset{t=1}{\sum }}\omega_{t}\eta_{i}=15.$$

In addition, the severity burden and multilevel involvement burden are assigned equal importance:


$$\lambda_{s}=0.5,\;\lambda_{m}=0.5.$$

Under this setting, the severity burden, multilevel burden, and final CSDS are computed as


$$B_{n}^{\text{sev}}=\frac{1}{15}\overset{5}{\underset{i=1}{\sum }}\overset{3}{\underset{t=1}{\sum }}s_{n,i}^{\left(t\right)},$$

$$B_{n}^{\text{mul}}=\frac{1}{15}\overset{5}{\underset{i=1}{\sum }}\overset{3}{\underset{t=1}{\sum }}m_{n,i}^{\left(t\right)},$$

$$c_{n}=0.5B_{n}^{\text{sev}}+0.5B_{n}^{\text{mul}}.$$

#### 2.5.1 Case A: focal low-burden degeneration

This case represents a patient with only one moderate lesion at L4/L5 and no other visible degeneration. Specifically, only the spinal canal stenosis grade at L4/L5 is set to 1, whereas all other grades are 0. Therefore, the total normalized severity is

$$\sum \overset{\left(t\right)}{\underset{n,i}{s}}=0.5,$$

and the number of clinically significant findings is

$$\sum \overset{\left(t\right)}{\underset{n,i}{m}}=1.$$

$$B_{A}^{\text{sev}}=\frac{0.5}{15}\approx 0.033,\;B_{A}^{\text{mul}}=\frac{1}{15}\approx 0.067,$$

and the final patient-level score is

$$c_{A}=0.5\times 0.033+0.5\times 0.067=0.05.$$

This very low score indicates that although a single moderate lesion is present, the overall degeneration burden remains limited. Hence, the patient would be considered *low-burden at the global patient level*.

#### 2.5.2 Case B: severe single-level degeneration with adjacent compensation

This case represents a typical lower-lumbar dominant degeneration pattern. At L4/L5, the patient has severe spinal canal stenosis, severe subarticular stenosis, and severe neural foraminal narrowing, i.e., grades (2, 2, 2). At L5/S1, the patient shows moderate spinal canal stenosis, moderate subarticular stenosis, and normal neural foraminal narrowing, i.e., grades (1, 1, 0). All other lumbar levels are normal. Therefore,

$$\sum s_{n,i}^{\left(t\right)}=3.0+1.0=4.0,$$

$$\sum m_{n,i}^{\left(t\right)}=3+2=5.$$

Thus,

$$B_{B}^{\text{sev}}=\frac{4.0}{15}\approx 0.267,\;B_{B}^{\text{mul}}=\frac{5}{15}\approx 0.333,$$

and the final score is

$$c_{B}=0.5\times 0.267+0.5\times 0.333=0.30.$$

Compared with Case A, the CSDS increases substantially because a focal but severe lesion triggers both a high local severity burden and multiple clinically significant findings. If a high-burden threshold of τ = 0.25 is adopted, this patient would be correctly identified as clinically high-burden.

#### 2.5.3 Case C: diffuse multilevel severe degeneration

This case represents a patient with extensive multilevel degeneration from L2/L3 to L5/S1, which is commonly observed in elderly patients with diffuse lumbar stenosis or degenerative deformity. The grades are set as follows:

L2/L3:(1,1,1), L3/L4:(2,2,1), L4/L5:(2,2,2), L5/S1:(2,1,1),

$$\sum s_{n,i}^{\left(t\right)}=1.5+2.5+3.0+2.0=9.0,$$

$$\sum m_{n,i}^{\left(t\right)}=3+3+3+3=12.$$

$$B_{C}^{\text{sev}}=\frac{9.0}{15}=0.60,\;B_{C}^{\text{mul}}=\frac{12}{15}=0.80,$$

and the final score is

$$c_{C}=0.5\times 0.60+0.5\times 0.80=0.70.$$

This case yields the highest score among the three examples, reflecting the fact that CSDS is sensitive not only to lesion severity but also to multilevel disease burden. Therefore, diffuse degeneration with broad segment involvement is naturally assigned a much higher patient-level burden score than focal degeneration.

#### 2.5.4 Interpretation

The three examples demonstrate that the proposed CSDS formulation produces a clinically plausible burden ordering:

$$c_{A}=0.05<c_{B}=0.30<c_{C}=0.70.$$

In other words, the score increases from focal low-burden degeneration to severe single-level disease and finally to diffuse multilevel degeneration. This behavior is desirable because it shows that the patient-level assessment is not determined by one isolated lesion alone, but instead reflects the joint effect of lesion severity and cumulative multilevel involvement. Therefore, the CSDS formulation provides an intuitive and interpretable bridge between segment-level grading results and patient-level burden-aware assessment.

## 3 Experiments and results

### 3.1 Datasets

To comprehensively evaluate the proposed anatomy-guided and context-aware framework, we used three publicly available datasets that serve different roles in the overall pipeline. To ensure a rigorous and leakage-free evaluation protocol, all experiments were conducted under **patient-level 5-fold cross-validation**. In each fold, the subjects were partitioned into training, validation, and test subsets without overlap, and all reported results are presented as the mean ± standard deviation across the five folds unless otherwise specified.

#### 3.1.1 VerSe and SPIDER datasets (Stage I anatomical pre-training)

The VerSe dataset was employed only as an *auxiliary* pre-training resource to improve the robustness of vertebral numbering and centroid estimation under anatomically challenging cases, such as transitional vertebrae and severe deformities (15). Since VerSe is a CT dataset rather than MRI, it was not used in the main grading benchmark. Instead, we used simple domain-randomization operations, including random intensity inversion and non-linear intensity transformation, to transfer coarse spatial priors from CT to the MRI setting. After this auxiliary initialization, the anatomical parsing module was fine-tuned on the SPIDER dataset, which provides voxel-level lumbar MRI annotations for vertebral bodies, intervertebral discs, and the spinal canal (9). In the final framework, the SPIDER-refined model was used to generate anatomy-guided masks, estimate disc centroids, determine lumbar motion segments, and support level-aligned ROI extraction.

#### 3.1.2 LumbarDISC dataset (Stage II–III grading and burden assessment)

The main experiments were conducted on the LumbarDISC dataset released in the context of the RSNA 2024 Lumbar Spine Degenerative Classification challenge (11–13). Following the official challenge setting, we retained the five condition-specific targets at each lumbar level: spinal canal stenosis (SCS), left neural foraminal narrowing (LFN), right neural foraminal narrowing (RFN), left subarticular stenosis (LSS), and right subarticular stenosis (RSS). These laterality-specific labels were preserved as independent segment-level targets during training and were further aggregated to compute the patient-level Clinically Significant Degeneration Score (CSDS) and associated burden-aware indicators.

#### 3.1.3 Grade distribution and imbalance reporting

Degenerative lumbar MRI labels were markedly imbalanced, with a predominance of normal/mild findings. To make this imbalance explicit, we report the severity distribution for each condition in Table 1. The overall dataset contained 9,945 level-wise labels for each condition, while the held-out test split reported here contained 1,989 level-wise labels per condition. Counts are reported at the level-wise condition-label level rather than at the patient level. In the held-out test split, the numbers of severe labels were 173 for spinal canal stenosis, 118 for left foraminal narrowing, 118 for right foraminal narrowing, 196 for left sub-articular stenosis, and 195 for right sub-articular stenosis.

**Table 1:** Grade distribution of the LumbarDISC level-wise grading labels used in this study.

Condition	Overall Normal/mild	Overall Moderate	Overall Severe	Held-out test Normal/mild	Held-out test Moderate	Held-out test Severe
Spinal canal stenosis	7,652 (76.94%)	1,428 (14.36%)	865 (8.70%)	1,530 (76.92%)	286 (14.38%)	173 (8.70%)
Left foraminal narrowing	8,110 (81.55%)	1,245 (12.52%)	590 (5.93%)	1,622 (81.55%)	249 (12.52%)	118 (5.93%)
Right foraminal narrowing	8,085 (81.30%)	1,270 (12.77%)	590 (5.93%)	1,617 (81.30%)	254 (12.77%)	118 (5.93%)
Left sub-articular stenosis	7,125 (71.64%)	1,840 (18.50%)	980 (9.85%)	1,425 (71.64%)	368 (18.50%)	196 (9.85%)
Right sub-articular stenosis	7,105 (71.44%)	1,865 (18.75%)	975 (9.80%)	1,421 (71.44%)	373 (18.75%)	195 (9.80%)

The overall columns summarize all level-wise labels, whereas the held-out test columns summarize the test split used for final reporting. Percentages are calculated within each condition and split.

### 3.2 Implementation details

The proposed framework was implemented in **PyTorch 2.0** and trained on a Linux workstation equipped with **two NVIDIA RTX 3090 GPUs** (24 GB VRAM each). To address the memory constraints imposed by the 3D architecture and the multi-level modeling strategy, we adopted a **stage-wise training scheme** together with Automatic Mixed Precision (AMP) and gradient accumulation.

#### 3.2.1 Stage I: anatomy-guided structural parsing

In the first phase, a 3D U-Net-style backbone, conceptually grounded in the U-Net/V-Net family of medical image segmentation architectures, was initialized on VerSe and subsequently fine-tuned on SPIDER (20, 21). The sagittal MRI input **X**^sag^ was resized to 320 × 320 × 16. The segmentation module was optimized using AdamW with an initial learning rate of 1 × 10^−4^ and a weight decay of 1 × 10^−4^. The segmentation objective was defined as

$$L_{\text{seg}}=0.7L_{\text{Dice}}+0.3L_{\text{CE}}.$$

Training was performed for 150 epochs with cosine annealing, and the checkpoint achieving the best validation Dice score was retained for downstream transfer. For reference, nnU-Net was used as a standard segmentation baseline during supplementary comparison (22), while SPINEPS was included as a representative recent MRI spine segmentation method (10).

#### 3.2.2 Stage II–III: segment-level grading and patient-level burden assessment

In the second phase, the segmentation backbone was frozen and used only for anatomical localization, centroid estimation, and structural biomarker extraction. Based on the predicted disc centroids **c**_*i*_, level-specific ROIs **R**_*i*_ of size 96 × 96 × 16 were dynamically cropped for each lumbar motion segment. The local feature encoder Φ_loc_ was implemented as a 3D ResNet-34 architecture (23, 24) and initialized with generic volumetric pre-training. The biomarker branch Φ_bio_ was implemented as a two-layer MLP. The spine-context module Φ_ctx_ consisted of a 2-layer Transformer encoder with token dimension *C*_*z*_ = 256 and 4 attention heads (25).

The grading and CSDS assessment branches were optimized using AdamW with an initial learning rate of 1 × 10^−4^, weight decay of 1 × 10^−4^, and cosine annealing over 150 epochs. The mini-batch size was set to 16 patients, corresponding to 16 × 5 = 80 segment-level tokens per forward pass. To maintain stable training under the 24 GB VRAM constraint, AMP was enabled and the gradient accumulation step was set to 4.

The overall training objective was formulated as

$$L=\alpha L_{\text{seg}}+\beta L_{\text{grade}}+\gamma L_{\text{CSDS}}+\delta L_{\text{cons}},$$

where the coefficients were empirically set to

α=1.0,  β=2.0,  γ=1.0,  δ=0.5.

The same validation-selected coefficients were used consistently in every fold; no coefficient was re-tuned after observing test-fold performance. To mitigate class imbalance in ordinal grading, the class-balancing terms $w_{t,k}^{+}$ and $w_{t,k}^{-}$ were computed from the inverse class frequencies within each training fold.

#### 3.2.3 Evaluation metrics

For LumbarDISC, we reported the official RSNA challenge metric, **Weighted Log Loss (W-Log Loss, lower is better)** (11–13), together with Macro F1-score and Cohen's Kappa (κ) for segment-level grading. For patient-level clinically significant degeneration assessment, we reported Accuracy and AUC. For Stage I supplementary evaluation, segmentation quality on SPIDER was assessed using Dice Similarity Coefficient (DSC) and Average Symmetric Surface Distance (ASSD), while vertebral numbering robustness on VerSe was evaluated using Identification Rate and global DSC (9, 15).

#### 3.2.4 Statistical comparison protocol

To determine whether the observed ablation gains were statistically supported, we performed paired two-sided statistical comparisons on out-of-fold predictions rather than deriving inferential results from the five fold-level mean values alone. AUC was compared using DeLong's test on patient-level out-of-fold probabilities (26). Macro F1, Cohen's κ, and W-Log Loss were compared using patient-cluster paired permutation tests with 10,000 resamples, using the patient as the resampling unit to account for correlation among multiple level-wise labels from the same subject. Multiple testing within each comparison family was controlled using Holm's sequential procedure (27). As shown in Table 2, the Full Framework significantly outperformed both the Context Encoder and Biomarker Fusion configurations across all evaluated metrics.

**Table 2:** Paired statistical comparison between the full framework and the two most relevant ablated configurations.

Comparison	Macro F1 *p*	Cohen's κ *p*	W-Log Loss *p*	AUC *p*
Full framework vs. context encoder	0.012	0.018	0.004	0.007
Full framework vs. biomarker fusion	< 0.001	< 0.001	< 0.001	< 0.001

*P* values were computed from paired out-of-fold predictions. AUC was compared using DeLong's test, whereas Macro F1, Cohen's κ, and W-Log Loss were compared using patient-cluster paired permutation testing.

### 3.3 Comparison with baseline methods

To verify the effectiveness of the proposed method, we compared it against several representative baseline architectures spanning global volumetric classification, patch-based lumbar MRI grading, and transformer-style volumetric modeling. Because prior lumbar spine studies differ substantially in preprocessing strategy, input modality, label definition, and cohort composition, direct comparison with published raw numbers would be methodologically unfair. Therefore, all baselines were re-implemented and trained from scratch on the same LumbarDISC folds under a unified protocol.

The compared methods include: (1) a global-volume classifier based on 3D ResNet-50 (23, 24); (2) a patch-based local analysis model inspired by SpineNet-style disc-centered lumbar MRI grading pipelines (5–7); (3) a transformer-based volumetric architecture derived from UNETR/Swin-UNETR-style modeling (28, 29); and (4) a multi-task spine transformer baseline re-implemented under our training protocol. In addition, the clinical relevance of these baseline families is supported by recent lumbar MRI studies that have explored deep learning for stenosis grading, disc pathology assessment, and model benchmarking in clinical cohorts (2, 3, 8).

**Figure 2:**
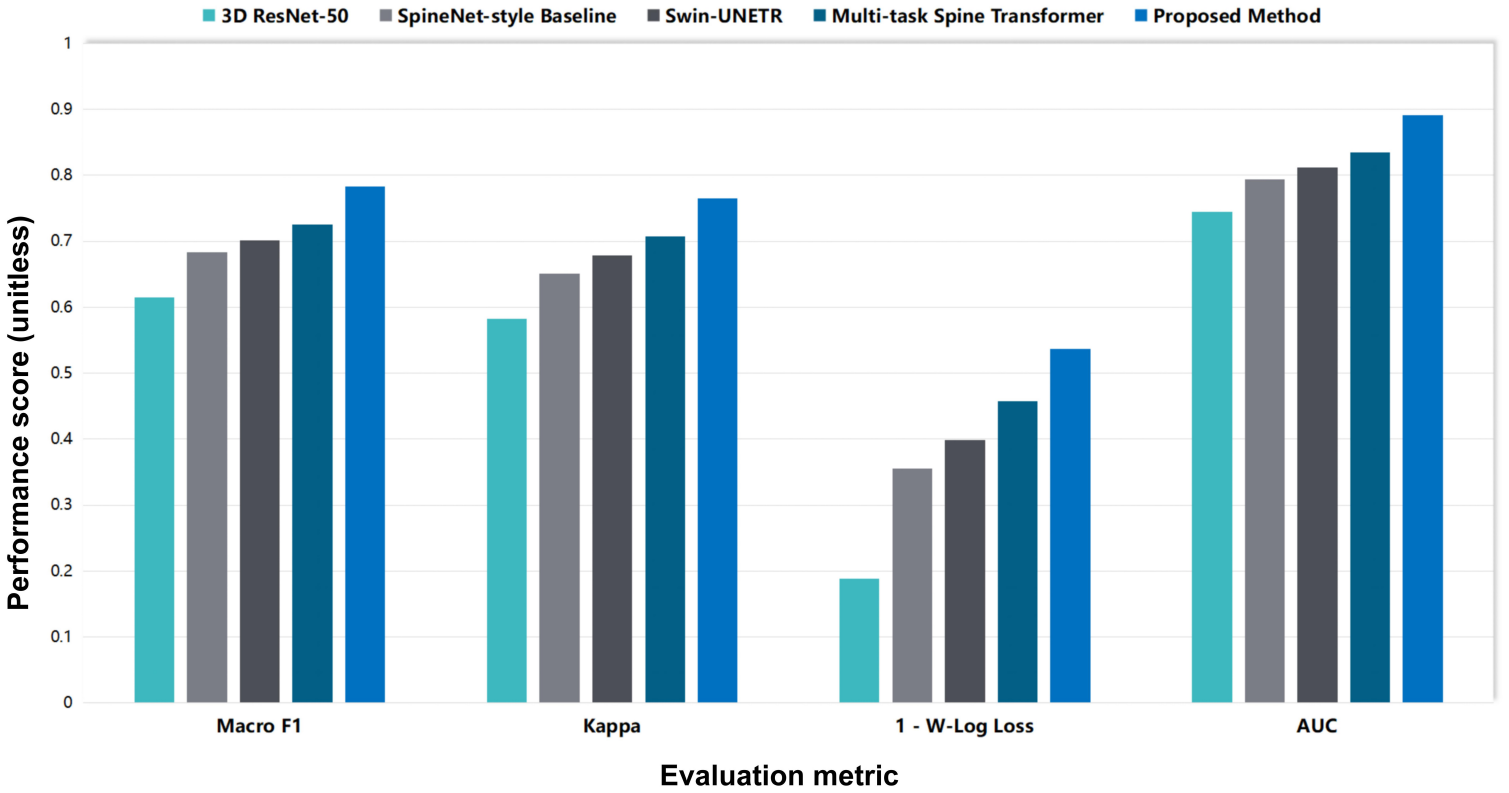
Comparison with representative re-implemented baseline architectures on the LumbarDISC benchmark. The plot summarizes Macro F1, Cohen's κ, 1 − W-Log Loss, and patient-level AUC under the same patient-level cross-validation protocol.

As shown in Figure 2 and Table 3, the proposed method consistently achieved the best performance across Macro F1, Cohen's Kappa, weighted log loss, Accuracy, and AUC. Compared with the strongest re-implemented baseline, our method reduced the official W-Log Loss from 0.542 ± 0.019 to 0.463 ± 0.018 and improved the patient-level AUC from 0.835 ± 0.014 to 0.891 ± 0.009. These improvements suggest that anatomy-guided level alignment, biomarker-aware fusion, and spine-context modeling provide complementary advantages beyond stronger feature extraction alone.

**Table 3:** Comparison with representative re-implemented baselines on the LumbarDISC dataset.

Method	Macro F1 ↑	Kappa (κ) ↑	W-Log Loss ↓	Accuracy ↑	AUC ↑
3D ResNet-50	0.612 ± 0.021	0.585 ± 0.024	0.810 ± 0.034	0.731 ± 0.020	0.742 ± 0.018
SpineNet-style baseline	0.683 ± 0.019	0.648 ± 0.021	0.644 ± 0.027	0.774 ± 0.016	0.789 ± 0.017
Swin-UNETR	0.701 ± 0.016	0.676 ± 0.018	0.602 ± 0.024	0.803 ± 0.014	0.810 ± 0.015
Multi-task spine transformer	0.724 ± 0.014	0.705 ± 0.016	0.542 ± 0.019	0.826 ± 0.013	0.835 ± 0.014
**Proposed method**	**0.783 ± 0.010**	**0.765 ± 0.012**	**0.463 ± 0.018**	**0.865 ± 0.011**	**0.891 ± 0.009**

All methods were trained and evaluated on the same patient-level folds. Lower W-Log Loss is better; higher values are better for all other metrics. Bold values indicate the best performance within each comparison metric.

From a methodological perspective, the superiority of the proposed framework can be attributed to three factors. First, the Stage I parser provides stable and interpretable level-specific ROI extraction, which substantially reduces anatomical ambiguity. Second, the biomarker branch explicitly preserves measurable geometric information that is not always robustly recoverable from image appearance alone. Third, the spine-context encoder captures the inter-level clinical dependency among adjacent lumbar segments, thereby producing a more coherent estimate of degeneration burden than independent level-wise classification.

### 3.4 Ablation study

**Figure 3:**
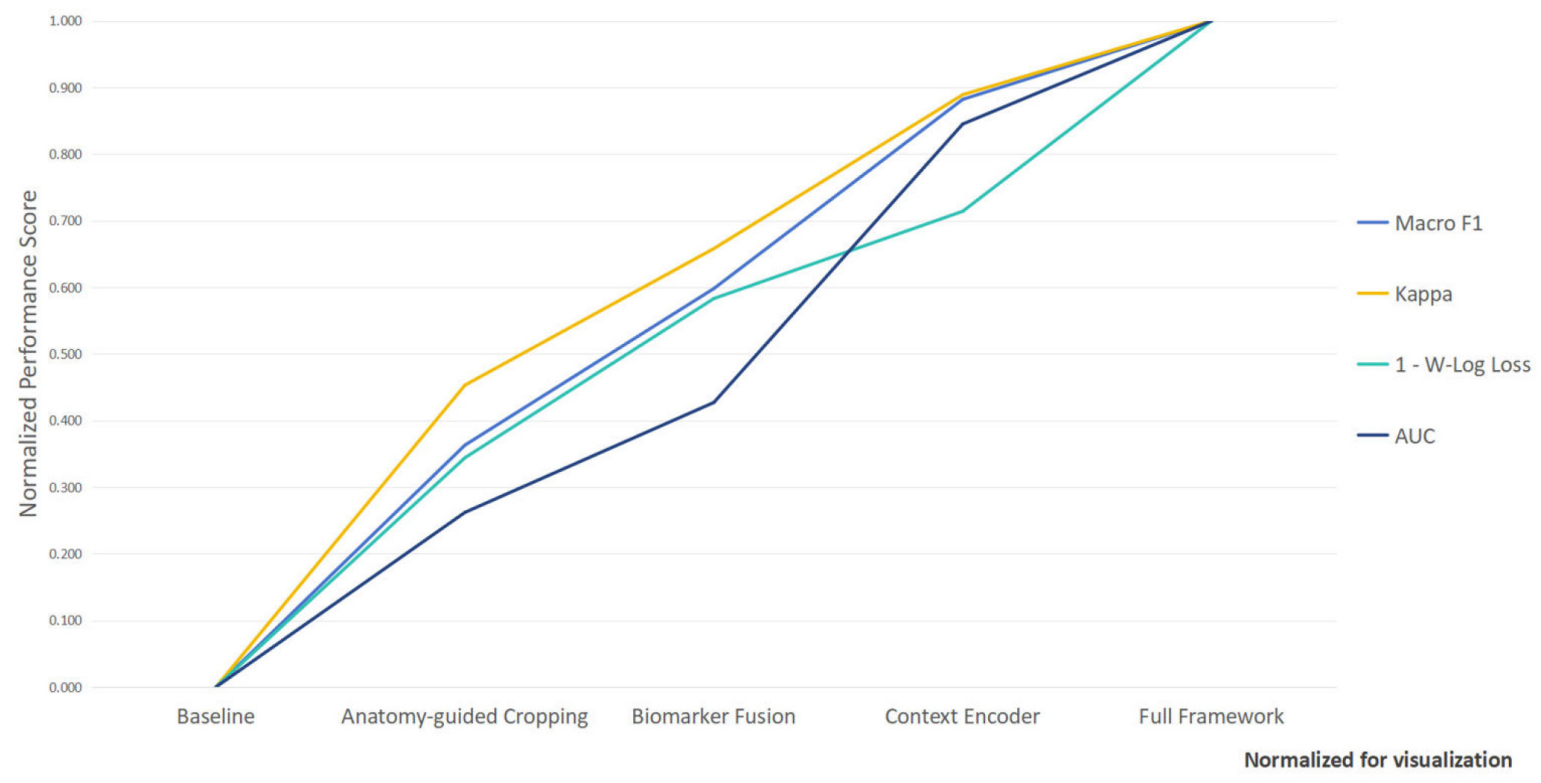
Progressive ablation trend of the proposed framework on the LumbarDISC dataset. The plot shows the normalized performance changes of Macro F1, Cohen's Kappa, 1 − W-Log Loss, and AUC as anatomy-guided cropping, biomarker fusion, spine-context modeling, and the full framework are progressively introduced. For visualization consistency, all metrics are normalized to [0, 1], with the baseline set to 0 and the full framework set to 1 for each metric. The results show a clear monotonic improvement across all evaluation criteria.

As shown in Figure 3, the proposed framework exhibits a clear progressive improvement as anatomy-guided cropping, biomarker fusion, and spine-context modeling are incrementally introduced. To isolate the contribution of each module, we conducted controlled ablation experiments under the same training protocol and cross-validation folds. The baseline configuration consisted of the local ROI encoder Φ_loc_ only with standard cross-entropy optimization. Starting from this baseline, we incrementally introduced anatomy-guided cropping, the biomarker branch, the spine-context encoder, and the consistency loss.

#### 3.4.1 Effectiveness of anatomy-guided structural parsing

Replacing centroid-aligned ROIs with loosely defined equidistant bounding boxes caused the grading Kappa to decrease from 0.765 ± 0.012 to 0.701 ± 0.019. This indicates that anatomy-guided spatial standardization substantially reduces the burden on the local encoder and allows it to focus more effectively on pathological texture and morphology.

#### 3.4.2 Impact of quantitative biomarker fusion

Removing the biomarker branch Φ_bio_ reduced the Macro F1 from 0.783 ± 0.010 to 0.742 ± 0.015. This confirms that explicit geometric priors, such as canal diameter and disc height, provide complementary evidence beyond latent image features, especially in borderline stenosis cases.

#### 3.4.3 Importance of the spine-context encoder

When the context encoder Φ_ctx_ was removed and all levels were classified independently, the patient-level AUC dropped from 0.891 ± 0.009 to 0.832 ± 0.016. This result highlights the necessity of modeling the anatomical and clinical dependence among adjacent lumbar levels.

#### 3.4.4 Influence of the consistency loss

Training without the consistency term *L*_cons_ degraded the official W-Log Loss from 0.463 ± 0.018 to 0.518 ± 0.021. This demonstrates that the consistency constraint acts as an effective regularizer by forcing the patient-level CSDS estimate to remain aligned with segment-level evidence.

**Figure 4:**
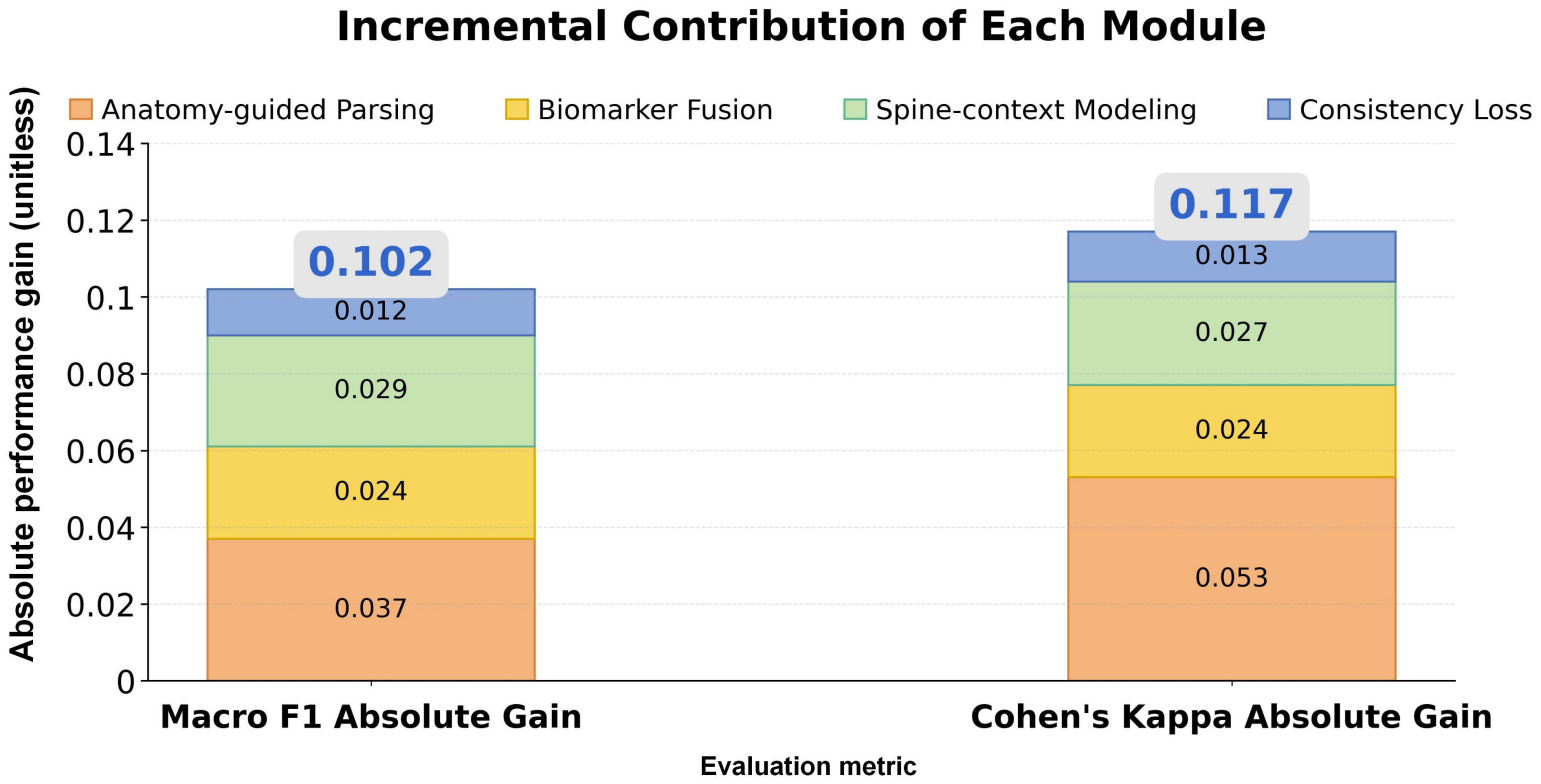
Incremental contribution of each module to the final performance gains. The stacked bars show the absolute gains on Macro F1 and Cohen's Kappa brought by anatomy-guided parsing, biomarker fusion, spine-context modeling, and consistency loss, respectively. The total gains from the baseline to the full framework are 0.102 for Macro F1 and 0.117 for Kappa, indicating that each module provides complementary improvements and that the final performance enhancement is achieved through cumulative integration rather than a single dominant component.

As illustrated in Figure 4 and summarized in Table 4, the gains brought by anatomy-guided parsing, biomarker fusion, spine-context modeling, and consistency loss are complementary, and their cumulative integration leads to the final improvement of the full framework.

**Table 4:** Ablation study of the proposed framework on the LumbarDISC dataset (5-fold cross-validation, mean ± std).

Configuration	Macro F1 ↑	Kappa (κ) ↑	W-Log Loss ↓	Accuracy ↑	AUC ↑
Baseline (Local ROI only)	0.681 ± 0.018	0.648 ± 0.022	0.655 ± 0.031	0.764 ± 0.017	0.788 ± 0.019
+ Anatomy-guided cropping	0.718 ± 0.015	0.701 ± 0.019	0.589 ± 0.028	0.791 ± 0.015	0.815 ± 0.014
+ Biomarker fusion (**q**_*i*_)	0.742 ± 0.015	0.725 ± 0.017	0.543 ± 0.022	0.807 ± 0.014	0.832 ± 0.016
+ Context encoder (Φ_ctx_)	0.771 ± 0.011	0.752 ± 0.014	0.518 ± 0.021	0.844 ± 0.012	0.875 ± 0.012
**Full framework (+ L_cons_)**	**0.783 ± 0.010**	**0.765 ± 0.012**	**0.463 ± 0.018**	**0.865 ± 0.011**	**0.891 ± 0.009**

Lower W-Log Loss is better; higher values are better for all other metrics. Bold values indicate the best performance within each comparison metric.

Overall, the ablation study shows a consistent and monotonic improvement as each proposed module is added. Importantly, the paired statistical comparisons in Table 2 further show that the Full Framework significantly improved over the Context Encoder configuration for Macro F1 (*p* = 0.012), Cohen's κ (*p* = 0.018), W-Log Loss (*p* = 0.004), and AUC (*p* = 0.007), and also significantly improved over the Biomarker Fusion configuration for all four metrics (*p* < 0.001). These results indicate that the performance gain of the full framework does not arise from a single architectural change or fold-level variability alone, but from the coordinated interaction of anatomy-guided localization, structured biomarker fusion, context-aware sequence modeling, and consistency-based regularization.

### 3.5 Discussion and interpretability

**Figure 5:**
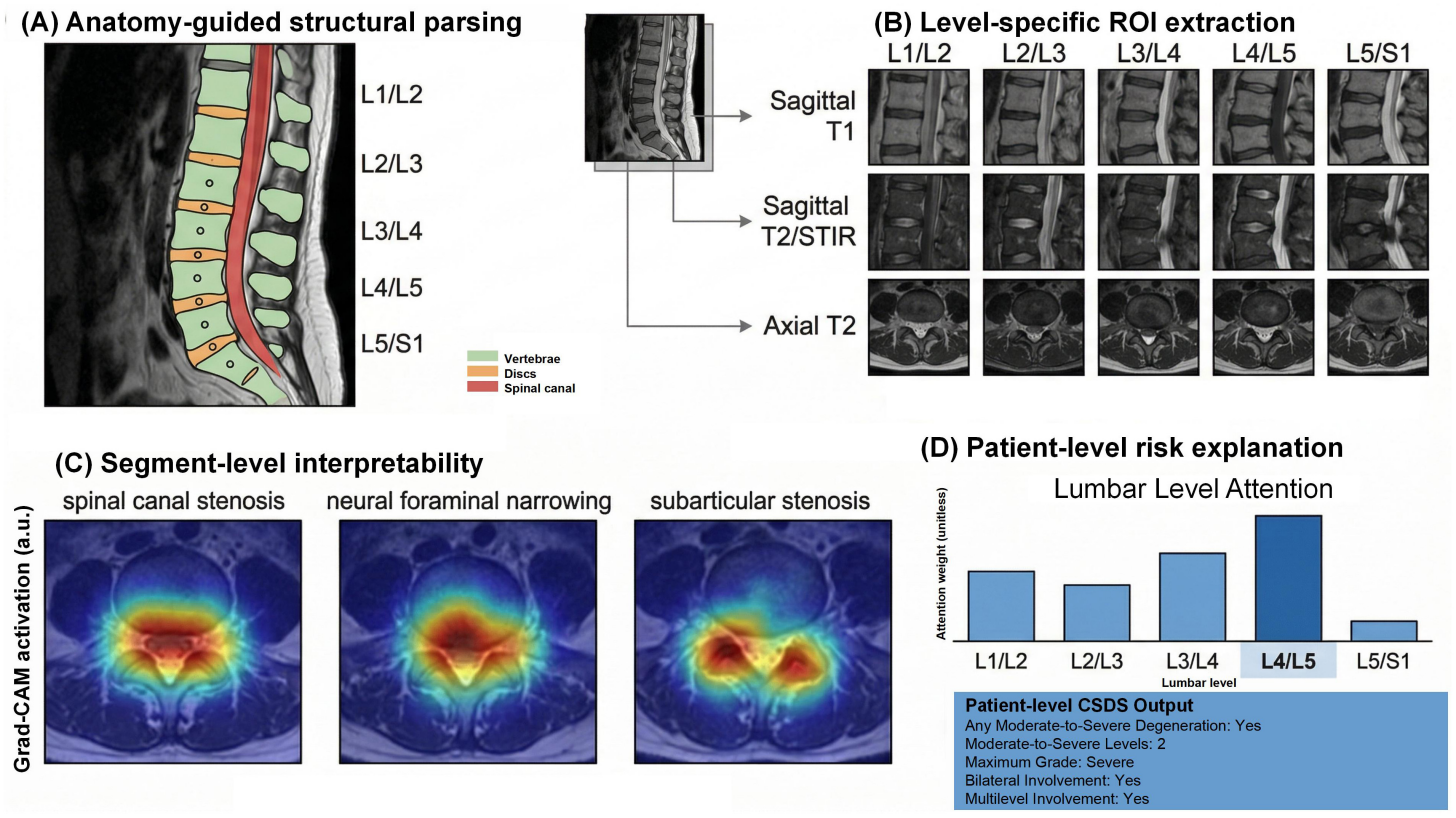
Qualitative visualization and interpretability analysis of the proposed framework. **(A)** An illustrative anatomy-guided structural parsing result, showing vertebral bodies, intervertebral discs, and the spinal canal together with lumbar level localization from L1/L2 to L5/S1. **(B)** Anatomy-guided extraction of level-specific multi-sequence MRI patches, including sagittal T1, sagittal T2/STIR, and axial T2 views for each lumbar motion segment. **(C)** Representative Grad-CAM visualizations for spinal canal stenosis, neural foraminal narrowing, and subarticular stenosis, showing that the model focuses on clinically relevant pathological regions. **(D)** Patient-level CSDS aggregation visualization using lumbar level attention weights and the corresponding Clinically Significant Degeneration Score (CSDS)-based output. These weights are presented as aggregation weights only and are not interpreted as causal feature-importance maps.

As shown in Figure 5, the proposed framework provides interpretable evidence at both the segment and patient levels, including anatomy-guided structural parsing, level-specific ROI alignment, Grad-CAM-based lesion visualization, and burden-aware patient-level explanation. The experimental results support our central hypothesis that automated lumbar MRI analysis should respect both *local anatomical boundaries* and *global inter-level continuity*. The proposed framework is effective because it mirrors the radiological reading process: first localizing the target level, then evaluating measurable structural changes, and finally integrating multi-level findings into an overall estimate of clinically significant degeneration burden.

Importantly, the model also provides interpretable intermediate evidence rather than functioning as a pure black box. The Stage I parser explicitly outputs vertebral, disc, and canal masks, which can be visually inspected and quantitatively validated. At the segment level, Grad-CAM activation maps (30) visualize the local regions that contribute most strongly to each grade assessment. At the patient level, the attention weights in

$$\text{g}=\overset{N}{\underset{i=1}{\sum }}\alpha_{i}{\text{h}}_{i}$$

are reported only as level-wise aggregation weights rather than as definitive causal feature-importance maps. Therefore, the evidence supporting the spine-context encoder is based primarily on the controlled ablation experiments and paired statistical comparisons, with Grad-CAM and attention visualization serving as auxiliary interpretability tools rather than standalone proof of context-driven correction. A fully auditable case-level demonstration of context-corrected local grading would require the same held-out patient, lumbar level, ground-truth grade, local-only and context-enhanced predicted probabilities, adjacent-level context, and corresponding image evidence to be linked within a single reproducible case package.

From a clinical-use perspective, these intermediate outputs should be treated as an audit trail rather than as independent decision rules. The final segment-level grades and the final CSDS represent the primary model outputs for decision support, whereas the anatomical masks, quantitative biomarkers, Grad-CAM maps, and level-wise attention weights help clinicians judge whether those outputs are anatomically plausible and concordant with the visible MRI findings. When the final CSDS and the intermediate evidence appear discordant, clinicians should first inspect the relevant MRI slices, segmentation masks, ROI alignment, and local heatmaps; the attention weights can prioritize which lumbar levels deserve closer review, but they should not override direct image assessment, patient context, or radiologist judgment.

These observations suggest that the proposed system is not only more accurate than the compared baselines, but also more suitable for clinically transparent decision support when attention is interpreted cautiously and in conjunction with ablation and statistical evidence. By jointly revealing *where* the most informative lesion resides, *which* anatomical measurements support it, and *how* the final burden score is formed, the framework improves the interpretability and trustworthiness of automated degenerative spine assessment.

### 3.6 Supplementary evaluation of Stage I structural parsing

Although the main objective of this study is segment-level grading and patient-level burden assessment on LumbarDISC, the reliability of the Stage I anatomical parser is a prerequisite for all downstream components. Therefore, we additionally report supplementary evaluation results on SPIDER and VerSe.

#### 3.6.1 Segmentation performance on SPIDER

The SPIDER dataset was used to assess voxel-level segmentation of lumbar vertebrae, intervertebral discs, and the spinal canal (9). We compared the proposed Stage I module against nnU-Net (22) and SPINEPS (10). As shown in Table 5, the proposed module achieved strong and stable performance across all structures, with the best overall boundary precision. In particular, the lower ASSD values indicate smoother and more anatomically plausible boundaries, which are beneficial for extracting reliable geometric biomarkers in the subsequent grading stage.

**Table 5:** Supplementary segmentation results on the SPIDER evaluation split.

Method	Vertebrae		Intervertebral discs (IVDs)		Spinal canal	
	DSC (↑)	ASSD (mm, ↓)	DSC (↑)	ASSD (mm, ↓)	DSC (↑)	ASSD (mm, ↓)
nnU-Net (baseline)	0.912 ± 0.015	0.45 ± 0.18	0.895 ± 0.021	0.62 ± 0.25	0.887 ± 0.031	0.51 ± 0.22
SPINEPS (reference)	0.933 ± 0.011	0.34 ± 0.14	0.918 ± 0.018	0.48 ± 0.19	0.902 ± 0.024	0.43 ± 0.16
**Proposed (stage I)**	**0.938 ± 0.009**	**0.31 ± 0.11**	**0.925 ± 0.014**	**0.41 ± 0.15**	**0.914 ± 0.019**	**0.38 ± 0.12**

Baseline values are listed for reference. Bold values indicate the best performance within each comparison metric.

#### 3.6.2 Robustness of vertebral numbering on VerSe

To further evaluate the robustness of vertebral identification under severe anatomical variation, we used VerSe as an auxiliary CT-based benchmark (15). Representative CT-side reference methods for this task include coarse-to-fine SpatialConfiguration-Net-based localization and more recent dual-branch segmentation designs (15, 31). We measured identification rate, defined as the percentage of correctly labeled vertebral centers, together with the global segmentation DSC. As shown in Table 6, the proposed pre-training strategy remained competitive with CT-specific methods while maintaining a high identification rate of 96.2%. This suggests that the spatial priors learned from VerSe generalized well enough to support reliable vertebral ordering in the downstream MRI pipeline.

**Table 6:** Supplementary vertebral identification and segmentation performance on VerSe.

Method	Modality focus	Identification rate (%) ↑	Global DSC ↑
V-Net (standard baseline)	CT only	88.5	0.902
SpatialConfig-Net	CT only	95.8	0.935
DBU-Net (reference)	CT only	**96.5**	**0.944**
**Proposed pre-training**	**Domain randomized**	96.2	0.941

Bold values indicate the best performance within each comparison metric.

Taken together, these supplementary results indicate that the proposed anatomical parser provides sufficiently reliable structural priors for the subsequent ROI extraction, biomarker calculation, and spine-context modeling stages.

## 4 General discussion and clinical implications

The present study demonstrates that anatomy-guided and context-aware modeling is an effective strategy for automated lumbar MRI assessment. On the public LumbarDISC benchmark, the proposed framework achieved superior segment-level grading and patient-level discrimination compared with all re-implemented baselines, while also providing interpretable intermediate evidence through anatomical masks, quantitative biomarkers, Grad-CAM maps, and level-wise attention. These findings support the view that lumbar degenerative assessment should not be treated as a purely local image classification problem, but rather as a structured multi-level reasoning task constrained by anatomy and biomechanics, which is also consistent with broader medical imaging evidence that multimodal and structure-aware learning can improve clinical relevance and model transparency (32–34).

Beyond quantitative performance, the clinical value of the framework lies in its transparency and workflow compatibility. The model mirrors the radiological reading process by first identifying the relevant lumbar level, then evaluating local structural abnormalities, and finally aggregating multilevel evidence into an overall burden-aware estimate. Such a design is more consistent with human-centered radiology than end-to-end black-box scoring because it enables visual inspection of anatomical parsing, review of quantitative structural evidence, and interpretation of patient-level outputs through level-wise contribution weights (16, 19, 35, 36). In practice, this may support more standardized grading, improved reporting consistency, and more transparent AI-assisted reading. From a sustainability perspective, such anatomy-guided localization and burden-aware summarization may reduce repetitive manual inspection of all lumbar levels, support more efficient structured reporting, and promote better use of radiological time and clinical resources. In this sense, the proposed framework contributes not only to interpretable and human-centered radiological AI, but also to workflow-conscious and resource-aware clinical practice.

Importantly, the patient-level output in this study should be interpreted as burden-aware risk assessment rather than true prognostic prediction. The CSDS target is derived from contemporaneous level-wise grading labels on cross-sectional public datasets rather than from longitudinal clinical outcomes. Therefore, the present framework estimates current clinically significant degenerative burden and relative burden status, but does not yet predict future progression, treatment response, or symptom evolution. This distinction is essential for correct clinical interpretation and for avoiding overstatement of translational readiness.

The study also has several limitations. First, the framework was primarily validated on retrospective public datasets, and its cross-center robustness in heterogeneous acquisition environments remains to be established. Second, although the anatomical parsing stage showed strong supplementary performance, residual localization errors may still affect downstream ROI extraction in highly atypical anatomy. Third, the current patient-level target does not incorporate symptoms, treatment history, electrophysiology, or longitudinal follow-up, which limits the model's ability to represent the full clinical complexity of lumbar degenerative disease. Fourth, although paired statistical testing has now been added for the model-comparison experiments, prospective reader-assistance studies are still needed before claiming workflow-level utility, as has also been emphasized in broader discussions of translational medical AI (19, 35, 36). Fifth, we did not introduce additional non-public institutional patient images or a new case report to illustrate a context-correction example, because no separate ethics approval or publication consent was obtained for publishing institution-specific patient images beyond the public datasets. For public-dataset experiments, a rigorously auditable context-correction figure would also require a complete visualization-ready package linking image crops, fold assignment, reference labels, local-only probabilities, context-enhanced probabilities, and adjacent-level evidence for the same held-out patient. The current revision therefore avoids an unverified anecdotal case and grounds the evidence for spine-context modeling in ablation and paired statistical testing.

Future work should therefore move in four directions. First, multi-center external validation should be performed to evaluate generalizability under scanner, protocol, and population shift. Second, uncertainty estimation and case-level confidence calibration should be incorporated to better support radiologist oversight. Third, structured-report supervision and multimodal fusion with symptoms or outcomes may allow a transition from burden estimation to clinically meaningful prognostic modeling (34). Fourth, prospective studies should assess whether the proposed system can reduce reporting variability, accelerate structured assessment, and improve the transparency of AI-assisted lumbar spine MRI interpretation. In addition, large-scale benchmark-driven training and foundation-style medical image models may further improve robustness under limited lumbar MRI annotations (22, 37, 38).

Overall, the proposed framework provides a technically strong and clinically interpretable foundation for automated lumbar degenerative disease analysis. By explicitly integrating anatomical priors, quantitative biomarkers, and multilevel context, it advances lumbar MRI assessment toward more transparent, standardized, and human-centered radiological AI.

## Data Availability

The original contributions presented in the study are included in the article/supplementary material, further inquiries can be directed to the corresponding author.
